# Mapping of Crowdsourcing in Health: Systematic Review

**DOI:** 10.2196/jmir.9330

**Published:** 2018-05-15

**Authors:** Perrine Créquit, Ghizlène Mansouri, Mehdi Benchoufi, Alexandre Vivot, Philippe Ravaud

**Affiliations:** ^1^ INSERM UMR1153, Methods Team Epidemiology and Statistics Sorbonne Paris Cité Research Center Paris Descartes University Paris France; ^2^ Centre d’Epidémiologie Clinique Hôpital Hôtel Dieu Assistance Publique des Hôpitaux de Paris Paris France; ^3^ Cochrane France Paris France; ^4^ Department of Epidemiology Columbia University, Mailman School of Public Health New York, NY United States

**Keywords:** review [publication type], crowdsourcing, health

## Abstract

**Background:**

Crowdsourcing involves obtaining ideas, needed services, or content by soliciting Web-based contributions from a crowd. The 4 types of crowdsourced tasks (problem solving, data processing, surveillance or monitoring, and surveying) can be applied in the 3 categories of health (promotion, research, and care).

**Objective:**

This study aimed to map the different applications of crowdsourcing in health to assess the fields of health that are using crowdsourcing and the crowdsourced tasks used. We also describe the logistics of crowdsourcing and the characteristics of crowd workers.

**Methods:**

MEDLINE, EMBASE, and ClinicalTrials.gov were searched for available reports from inception to March 30, 2016, with no restriction on language or publication status.

**Results:**

We identified 202 relevant studies that used crowdsourcing, including 9 randomized controlled trials, of which only one had posted results at ClinicalTrials.gov. Crowdsourcing was used in health promotion (91/202, 45.0%), research (73/202, 36.1%), and care (38/202, 18.8%). The 4 most frequent areas of application were public health (67/202, 33.2%), psychiatry (32/202, 15.8%), surgery (22/202, 10.9%), and oncology (14/202, 6.9%). Half of the reports (99/202, 49.0%) referred to data processing, 34.6% (70/202) referred to surveying, 10.4% (21/202) referred to surveillance or monitoring, and 5.9% (12/202) referred to problem-solving. Labor market platforms (eg, Amazon Mechanical Turk) were used in most studies (190/202, 94%). The crowd workers’ characteristics were poorly reported, and crowdsourcing logistics were missing from two-thirds of the reports. When reported, the median size of the crowd was 424 (first and third quartiles: 167-802); crowd workers’ median age was 34 years (32-36). Crowd workers were mainly recruited nationally, particularly in the United States. For many studies (58.9%, 119/202), previous experience in crowdsourcing was required, and passing a qualification test or training was seldom needed (11.9% of studies; 24/202). For half of the studies, monetary incentives were mentioned, with mainly less than US $1 to perform the task. The time needed to perform the task was mostly less than 10 min (58.9% of studies; 119/202). Data quality validation was used in 54/202 studies (26.7%), mainly by attention check questions or by replicating the task with several crowd workers.

**Conclusions:**

The use of crowdsourcing, which allows access to a large pool of participants as well as saving time in data collection, lowering costs, and speeding up innovations, is increasing in health promotion, research, and care. However, the description of crowdsourcing logistics and crowd workers’ characteristics is frequently missing in study reports and needs to be precisely reported to better interpret the study findings and replicate them.

## Introduction

Scientific research performed with the involvement of the broader public, the crowd, is attracting increasing attention from scientists and policy makers. Crowdsourcing uses the power of many, using the collective wisdom and resources of the crowd, to complete human intelligence tasks (ie, tasks that cannot be entirely automated and require human intelligence). Crowdsourcing is not a new concept and has often been used in the past as a competition to discover a solution. It originated in 1714 in England, where the British Government proposed £20,000 to anyone who could find a solution for calculating the longitudinal position of a ship [1], and then it was applied in a variety of fields such as astronomy, energy system research, genealogy and genetic research, journalism, linguistics, ornithology, public policy, seismology, and molecular biology [2].

Crowdsourcing currently involves a network of people, the “crowd workers,” responding to an open call and completing Web-based tasks of requesters [3]. These crowd workers provide a large wide of activities, especially via the internet, using specific platforms, but have no formal training in the topic of investigation [4]. They have access to the crowdsourcing websites from anywhere at times convenient for them. They carry out tasks posted by requesters, who accept or reject their work and may or not pay them for the work. Crowdsourcing has grown rapidly with the evolution of technology, with 2.3 billion internet users and 6 billion mobile phone subscribers [5]. The main Web platform for crowdsourcing is Amazon Mechanical Turk (MTurk), which was exploited by scientists 5 years ago. In 1 month—May 2016—23,000 people completed 230,000 tasks on their computers in 3.3 million min, corresponding to a total of more than 6 years of effort [6].

Crowdsourcing has several benefits. Crowdsourcing provides easy access to a potentially large pool of participants for a research problem, particularly for increasing the number of respondents for mining crowd data (eg, Web-based surveys) and active crowdsourcing (eg, data processing). It offers important time savings, in that a large number of contributors working in parallel reduces the time required to perform a fixed amount of work, mainly saving the elapsed time to collect data. Project organizers can lower the cost of labor inputs. By soliciting ideas from a large group of people through the internet, crowdsourcing can be used to speed up innovations, particularly those with challenges.

Crowdsourcing has been used primarily in nonmedical fields [7]. The Galaxy Zoo project successfully classified about 900,000 galaxies with the help of hundreds of thousands of Web-based volunteers [8]. The eBird project collected more than 48 million bird observations from more than 35,000 contributors [9]. The fields of research using Amazon MTurk are psychology, marketing, management, business, political science, computer science (improvement of artificial intelligence software, for example, by naming objects to help the computer identify the content of a photograph), and neuroscience [6]. Crowdsourcing is becoming the center of attention of the scientific community and researchers needing to obtain data from any domain.

Crowdsourcing represents a great opportunity in health and medical research. As mentioned by Swan [10], crowdsourced health research studies are the nexus of 3 contemporary trends: “citizen science,” crowdsourcing, and Medicine 2.0. Medicine 2.0 or Health 2.0 refers to the active participation of individuals in their health care, particularly using Web 2.0 technologies.

Crowdsourcing is not limited to health research but can also be used in health promotion or health care. Crowdsourcing could be a great way to solve a specific scientific mission that cannot be entirely automated and requires human intelligence in these 3 health categories. However, mapping of crowdsourcing use in health is needed to describe all its applications and to detail specificities, so that health researchers can assess whether they can use this approach in their research.

The aim of the study was to map the different applications of crowdsourcing used in health to outline the fields of health that are using crowdsourcing and the type of crowdsourced tasks involved. We also describe the logistics of crowdsourcing and the characteristics of crowd workers.

## Methods

### Design

We conducted a systematic review to identify studies using crowdsourcing in health. We uploaded a prespecified protocol to a publicly accessible institutional Website (Multimedia Appendix 1) and followed standard procedures for systematic reviews and reported processes and results according to the Preferred Reporting Items for Systematic Reviews and Meta-Analyses guidelines [11].

### Criteria for Considering Studies for This Review

The inclusion criteria were as follows:

1. Studies reporting on health, considering the definition proposed by Prpic [12], with the activities of the 3 categories of health:
  - Health promotion: disease detection and surveillance, behavioral interventions, health literacy, and health education
  - Health research: pharmaceutical research, clinical trials and health experiment methodology, and improving health care research knowledge
  - Health maintenance (here “health care”): patient- or physician-related, diagnostics, medical practice, and treatment support.
2. Studies conducted with a crowdsourced population: workers are recruited by crowdsourcing (ie, recruited via a website [labor markets such as Amazon MTurk or Crowdflower] or an open call to a large audience using internet-related technologies [eg, scientific games or community challenges with dedicated platforms]) [13]. Studies can refer either to a feasibility study (can crowdsourcing be used for a specific task?) or to the use of crowdsourcing to supply data that support a finding in some research activity.

We excluded studies considering structural and molecular biology (eg, studies reporting Web-based games to manipulate the 3D structures of proteins or moving colored blocks representing different nucleotide sequences).

### Search Method for Identification of Studies

We performed an electronic search of MEDLINE via PubMed and EMBASE to identify all reports published from inception to March 30, 2016, with no restriction on date, language, study design, or publication status (published papers or conference abstracts). All databases were searched using both controlled vocabulary (namely, MeSH terms in MEDLINE and Emtree terms in EMBASE) and a wide range of free-text terms. Indeed, crowdsourced health studies may be a blend of crowdsourcing and citizen science (ie, nonprofessionally trained individuals conducting science-related activities); these terms can be used interchangeably and so were included in our search equation. We used different terms referring to crowdsourcing, citizen science, and Web platforms. The search strategy used to search MEDLINE and EMBASE is in Multimedia Appendix 2. We also screened ClinicalTrials.gov (search strategy in Multimedia Appendix 3) and the reference lists of previous systematic reviews [5,10] and selected papers to identify additional studies.

### Selection of Studies

Two reviewers (PC and GM) independently examined each title and abstract identified to exclude irrelevant reports. The 2 reviewers then independently examined full-text articles to determine eligibility. Disagreements were discussed to reach consensus. We documented the primary reason for exclusion of full-text articles. For ClinicalTrials.gov, only studies with posted results were included.

### Definition of the Crowdsourcing Tasks

We used the classification described by Ranard [5] with 4 tasks of crowdsourcing: (1) problem-solving: to propose empirical solutions to scientific problems; (2) data processing: to perform several human intelligence microtasks to provide in total an analysis of a large amount of data; (3) surveillance or monitoring: to find and collect information into a common location and format such as the creation of collective resources; and (4) surveying: to answer a Web-based survey. Surveillance or monitoring and surveying belong to mining crowd data described by Khare [4] and are defined as data collected and analyzed by crowd workers for the knowledge discovery process. Problem-solving and data processing belong to active crowdsourcing, which refers to crowd workers recruited to solve scientific problems.

### Data Extraction and Management

The data were extracted from reports by the two reviewers (PC and GM) who used a standardized data extraction form (provided with the protocol as Multimedia Appendix 1). Disagreements were discussed to reach consensus. From each study, we extracted the following characteristics.

#### Publication Characteristics of the Study

Publication characteristics of the study were as follows: *Journal Citation Reports* categories (ie, general medicine and health care science, biomedical informatics and technology, or medical specialty journals); impact factor (Clarivate Analytics); average journal impact factor percentile from *Journal Citation Reports* (classified in four categories: >90th percentile, 70th-90th percentile, <70th percentile, and not indexed); and year of publication.

#### Characteristics of Crowdsourcing Applications in Health

The following characteristics of crowdsourcing applications were extracted:

1. We determined the category of health the study referred to (health promotion, research, or care [12]) and health field (eg, public health, surgery, oncology [details in Multimedia Appendix 4]).
2. We classified the tasks into 1 of the 4 categories of crowdsourcing tasks defined: problem-solving, data processing, surveillance or monitoring, and surveying.
3. We determined whether the study was led by researchers (ie, a traditional study led by institutionally trained researchers) or by participants (ie, studies designed and operated by patients or citizen scientists) [10].

#### Logistics of Crowdsourcing and Characteristics of Crowd Workers

Considering the logistics of crowdsourcing and characteristics of crowd workers, the following points were extracted:

1. We defined how the crowdsourcing was applied: whether a large task was divided into microtasks and distributed to workers [13] or whether the same task—a high-difficulty task called a megatask, such as a challenge—was given to several groups of workers [14].
2. We extracted the type of platform used (labor markets, scientific games, mobile phone apps, social media, or community challenges with dedicated platforms) [13]; whether monetary incentives were offered and their amount; the time to perform the task; whether a data quality validation was performed; whether the task performed by the crowd workers was compared with that performed by experts (which corresponds to a feasibility study).
3. We extracted the number of crowd workers, the median age, the proportion of women, their status (eg, researchers, physicians, and students), their geographic location, their motivations, whether a skill set was required to perform the task, and whether they had to undergo training and pass a qualification test to be recruited.
4. We also assessed the proportion of studies not reporting all these data.

### Analysis

The analysis was descriptive. Data are summarized as number (%) for qualitative variables and median (Q1-Q3) for continuous variables. All analyses involved the use of R v3.0.2 (R Foundation for Statistical Computing, Vienna, Austria) [15].

## Results

### Systematic Literature Search

The flow of study selection is in Multimedia Appendix 5. Briefly, the electronic search yielded 2354 references; 326 were selected for further evaluation, and 202 studies were included (182 published papers and 20 conference abstracts [3,16-216]).

**Figure 1:**
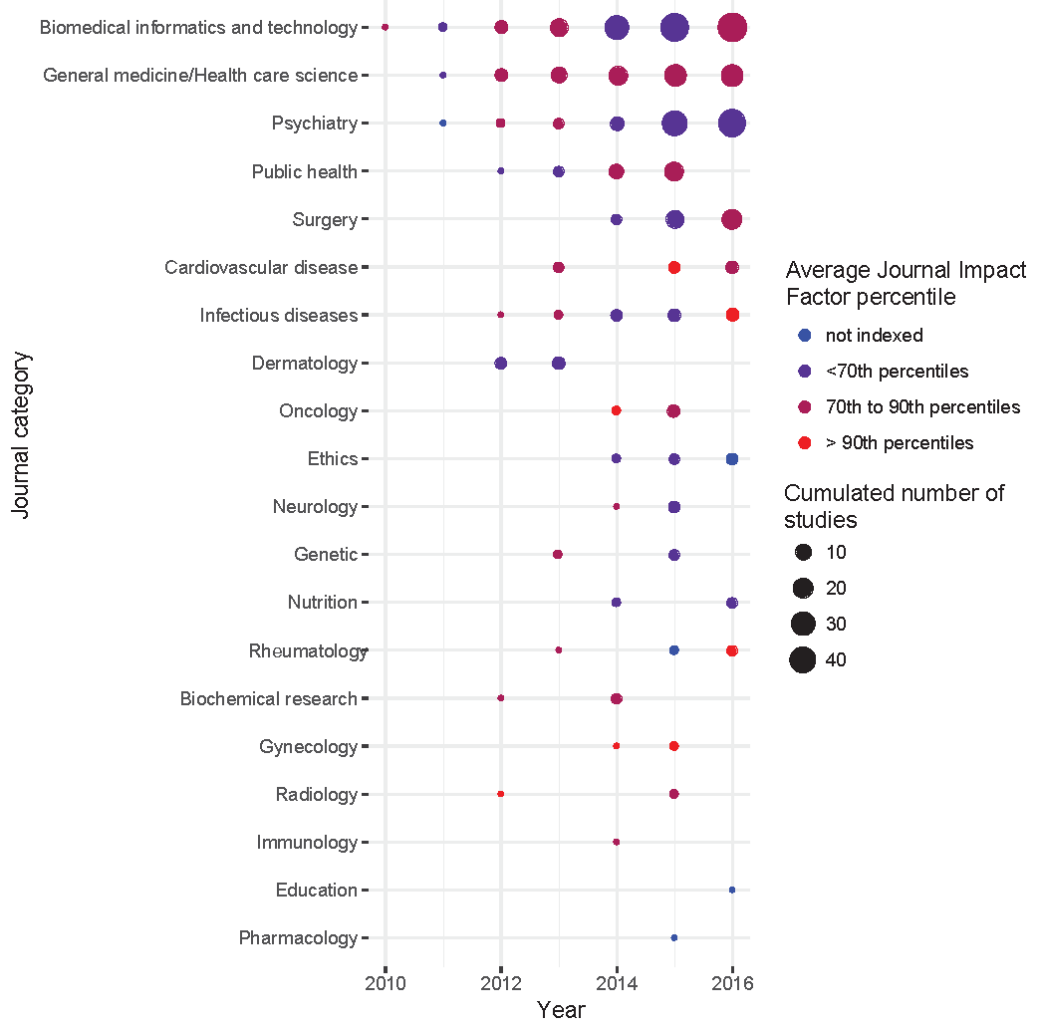
Publication characteristics of included studies. Two-thirds of the studies have been published in one of the 18 medical specialty journals, covering almost all medical fields showing the widespread use of crowdsourcing, and sometimes in a journal with very high relative impact factor.

More than half of the included studies (108/202, 53.5%) were published during the last 2 years. The median impact factor of the journals of publication was 3.2 (Q1-Q3: 2.1-3.5); for 42/202 studies (20.8%), reports were published in a journal with very high relative impact factor (>90th percentile of journal impact factors averaged across journal categories). Reports for two-thirds of studies (129/202) were published in medical specialty journals and for one-fourth (50/202) in biomedical informatics and technology journals. All these publication characteristics are in Figure 1. A total of 9 studies corresponded to randomized controlled trials, only 1 with results posted on ClinicalTrials.gov.

### Mapping of Crowdsourcing Applications in Health

**Figure 2:**
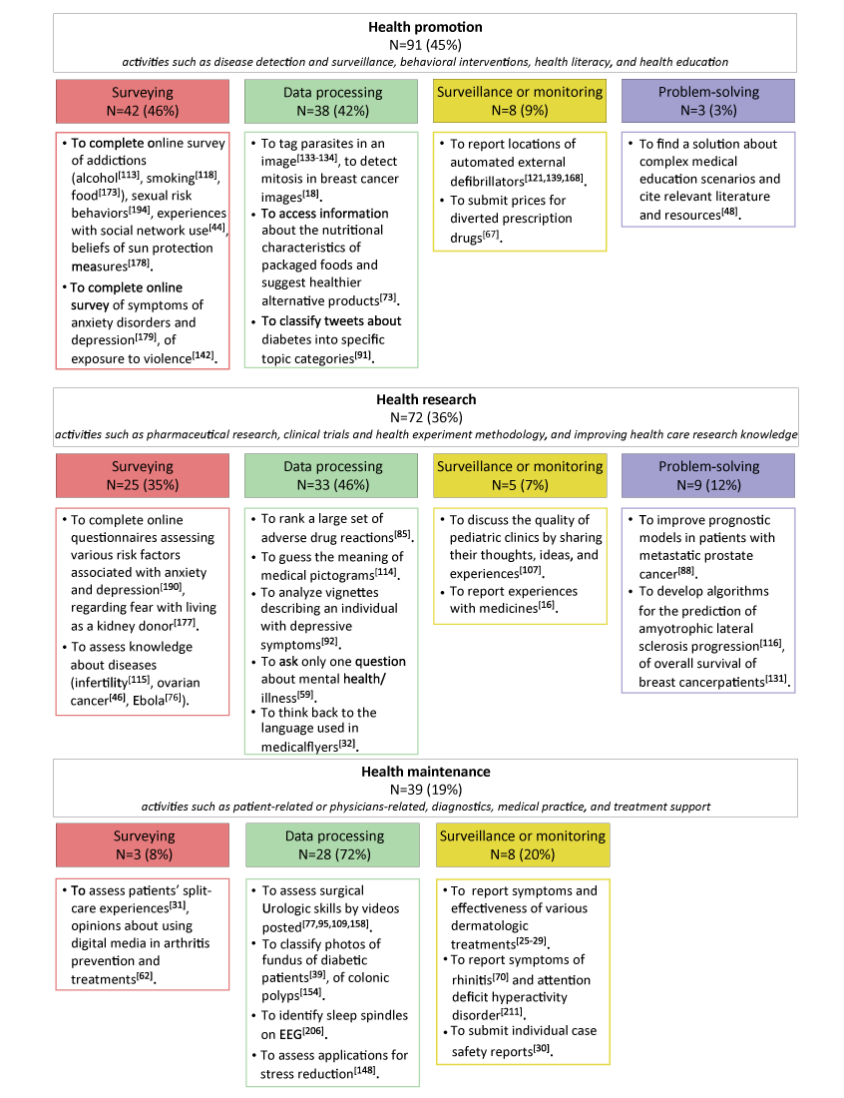
Examples of crowdsourced tasks according to health category. EEG: electroencephalography.

Crowdsourcing applications were more frequent in studies of health promotion (91/202, 45.0%) and health research (72/202, 35.7%) than health care (39/202, 19.3%). More than half of the studies concerned active crowdsourcing (data processing (99/202, 49.0%) and problem-solving (12/202, 5.9%)) and 45% of the studies were about mining crowd data (surveying (70/202, 34.6%) and surveillance or monitoring (21/202, 10.4%)). Examples of crowdsourced tasks by health category are provided in Figure 2.

Almost 50% of the studies related to health promotion used surveys to conduct their research compared with studies related to health care, which used mainly data processing activity. All included studies were led by researchers.

**Figure 3:**
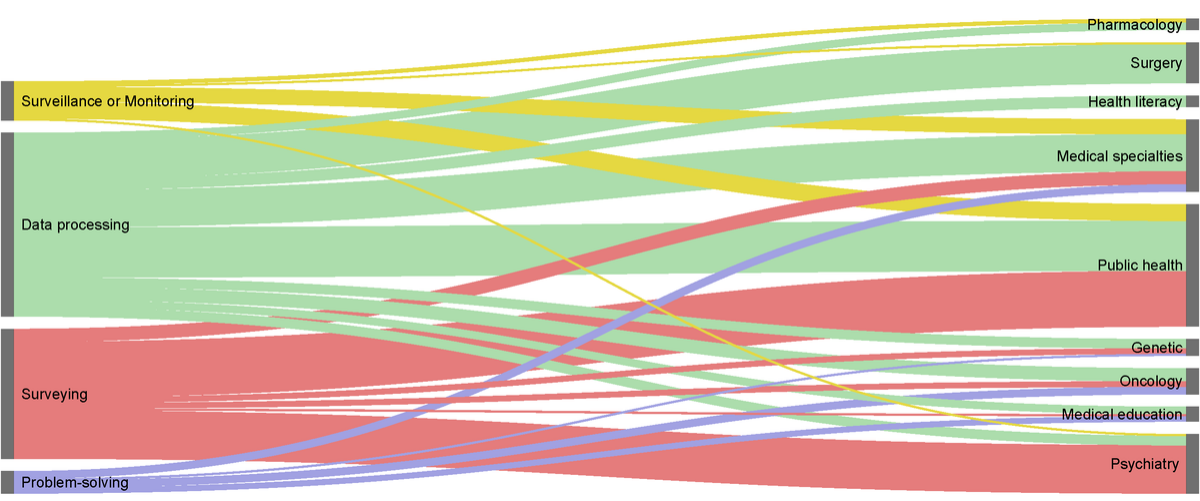
Mapping of crowdsourcing applications in health. Sankey diagram representing the distribution of medical fields applying crowdsourcing for each of the 4 types of task. Width of links is proportional to the number of studies. Medical specialties: anatomopathology (n=3), cardiology (n=5), dermatology (n=5), endocrinology (n=1), gynecology (n=2), infectiology (n=6), nephrology (n=1), neurology (n=7), pediatrics (n=2), pneumology (n=3), radiology (n=2),and rheumatology (n=2).

In Figure 3, we provide a mapping of crowdsourcing applications in health, detailing the medical fields applied to each type of task.

#### Data Processing

One-fourth of studies (27/99) involved public health, one-fifth (21/99) involved surgery, and one-fifth involved medical specialties (20/99). For example, in the Ghani et al study, published in 2016, crowd workers used the Global Evaluative Assessment of Robotic Skills tool to assess surgical skill in a video recording of a nerve-sparing robot-assisted radical prostatectomy [77].

#### Surveying

A total of 43% of studies (30/70) involved public health, and 37% (26/70) involved psychiatry. In the Stroh et al study, published in 2015, crowd workers completed a questionnaire related to public views on organ donation for people who need transplantation because of alcohol abuse [192]. The survey measured attitudes on liver transplantation in general and early transplantation for this patient population.

#### Surveillance or Monitoring

A total of 43% of studies (9/21) concerned public health and 24% (5/21) concerned dermatology. In the Merchant et al study, published in 2012, during 2 months, crowd workers had to locate, photograph, and submit the most eligible automated external defibrillator in Philadelphia [168].

#### Problem-Solving

One-third of studies (4/12) concerned oncology and one-fourth (3/12) concerned medical education. In the Margolin et al study, published in 2013, crowd workers were challenged during 6 months to develop computational models that predict overall survival of breast cancer patients based on clinical information [131].

#### Reporting of the Logistics of Crowdsourcing and Crowd Workers’ Characteristics

For data processing and surveillance or monitoring, a large task was divided into microtasks and distributed to crowd workers. For problem-solving, a megatask was given to several groups of crowd workers. We identified 7 challenges in our sample. A Web platform was used in 190/202 studies (94.1%), of which 133/190 (70.0%) were labor markets (eg, Amazon MTurk; Table 1).

Crowd workers’ characteristics and crowdsourcing logistics were poorly reported. Reports for almost one-fourth of studies (47/202) did not mention monetary incentives, and for two-thirds of studies (130/202), the time to perform the task was not mentioned. Crowd workers’ characteristics were frequently missing: age and gender were not reported for about 60% of the studies (128/202 and 105/202, respectively), and crowd workers’ location was not reported for one-fourth of the studies (50/202).

For 109/202 studies (53.9%), reports mentioned monetary incentives, mainly less than US $1 to perform a task. When reported, the time needed to perform the task was mostly less than 10 min (42/72, 58% of studies). For one-fourth of studies (54/202), reports mentioned using data quality validation, mainly by attention check questions (19/54, 35%) or by replicating the task by several crowd workers (16/54, 30%). About one-fifth of studies (36/202) compared crowd workers’ performance with that of experts (corresponding in these cases to a feasibility study), mainly for evaluating surgical skills (15/36, 42%).

The number of crowd workers was reported for 176 studies (87.1%), and the size of the crowd varied from 5 to about 2 million, with median 424 (first and third quartiles Q1-Q3: 167-802; Table 2). When specified, crowd workers’ median age was 34 years (Q1-Q3: 32-36) and 55% were men. Crowd workers were recruited nationally in 93/152 studies (61.2%), mainly the United States (83/93, 89%).

**Table 1:** Logistics of crowdsourcing in systematic review studies.

Characteristics			Statistics (N=202)
**Type of platform used**, **n (%)**			
	**Web platform**		190 (94.0)
		Labor markets^a^	133 (70.0)
		Social media^b^	10 (5.3)
		Labor markets and social media	2 (1.0)
		Community challenge	7 (3.7)
		Scientific games	6 (3.2)
		Other websites	32 (16.8)
	Mobile apps		12 (6.0)
**Monetary incentives to perform a task,** **n (%)**			
	Not reported		47 (23.2)
	**Yes**		109 (54.0)
		≤US $0.1	15 (13.8)
		US $0.2 to 0.5	30 (27.5)
		US $0.6 to 1	23 (21.1)
		>US $1	23 (21.1)
		Amount not specified	18 (16.5)
	No		46 (22.8)
**Time to perform a task, n (%)**			
	Not reported		130 (64.4)
	**Reported**		72 (35.6)
		≤1 min	17 (24)
		2 to 10 min	25 (35)
		11 to 30 min	21 (29)
		30 min to 1 hr	9 (12)
**Data quality validation, n (%)**			
	Not reported		148 (73.3)
	**Yes**		54 (26.7)
		Task replication by several CWs^c^	16 (30)
		Attention check questions	19 (35)
		Discriminative questions	12 (22)
		Limited timing for the task	4 (7)
		Not specified	3 (6)
**CW performance compared with experts, n (%)**			
	Yes		36 (17.8)

^a^Amazon MTurk, Crowdflower.

^b^Mainly Facebook, Twitter, LinkedIn, and Curetogether.

^c^CW: crowd worker.

**Table 2:** Characteristics of crowd workers.

Characteristics				Statistics (N=202)
**Size of the crowd**				
	Median (Q1-Q3)			424 (167-802)
	Not reported, n (%)			26 (12.9)
	**Reported, n (%)**			176 (87.1)
		<100		20 (11.4)
		100-499		78 (44.3)
		500-999		41 (23.3)
		1000-4999		25 (14.2)
		5000-10,000		7 (4.0)
		>10,000		5 (2.8)
**Geographic location, n (%)**				
	Not reported			50 (24.8)
	Reported			152 (75.2)
		International		59 (38.8)
		**National**		93 (61.2)
			United States	83 (89.2)
			Canada	2 (2.2)
			The Netherlands	3 (3.2)
			Other^a^	5 (5.4)
**Age**				
	Median (Q1-Q3)			34 (32-36)
	Not reported, n (%)			128 (63.4)
	Reported, n (%)			74 (36.6)
**Gender**				
	Not reported, n (%)			105 (52)
	Mean proportion of men (%)			55.0
**Status, n (%)**				
	Not reported			61 (30.2)
	**Reported**			141 (69.8)
		Anyone		51 (36.2)
		People graduated from college		35 (24.8)
		People with specificities		19 (13.5)
		Patients		14 (9.9)
		Medical or health care providers		9 (6.4)
		Researchers		8 (5.7)
		Students		5 (3.5)
**Skill set required, n (%)**				
	Not specified			128 (63.4)
	**Yes**			74 (36.6)
		Master qualification^b^		44 (60)
		Speak English		14 (19)
		Scientific background		8 (11)
		Medical background		8 (10)
**Qualification test, n (%)**				
	Not reported			34 (16.8)
	Yes			26 (12.9)
	No			142 (70.3)
**Training of workers, n (%)**				
	Not reported			31 (15.3)
	Yes			22 (10.9)
	No			149 (73.8)

^a^India, Australia, Israel, China, and South Korea.

^b^Defined as “consistently completing *human intelligence tasks* of a certain type with a high degree of accuracy across a variety of requesters.”

The motivations of crowd workers were recorded for 5/202 studies (2.5%) and included fun, curiosity, altruism, compensation, contribution to an important cause, personal reasons, research education, and advancing science [82,139,168,183,193]. A skill set was required in 74/202 studies (36.7%); for 60%, this involved previous experience in crowdsourcing. For two-thirds of studies (128/202), a specific skill set required was not specified. For only 12.8% of studies, the Web-based tasks (26/202) required passing a qualification test, and for 10.9%, (22/202), they required training.

## Discussion

### Principal Findings

In this systematic review of the use of crowdsourcing in studies of health promotion, research, and care, we included 202 studies, mainly published in the last 2 years with for one-fifth of a publication in a journal with very high relative IF. Data processing was the most frequent type of task used (mainly in public health and surgery), followed by surveying (public health and psychiatry), then surveillance or monitoring (public health and dermatology), and finally problem-solving (oncology). Labor market platforms (Amazon MTurk) were mainly used. The description of crowdsourcing logistics and crowd workers’ characteristics were frequently missing from reports. When reported, the median size of the crowd was less than 500; crowd workers’ median age was around 34 years and 55% were men. Crowd workers were mainly recruited in the United States. A previous experience in crowdsourcing was required in about 60% of the studies, whereas passing a qualification test or training was only needed in about 12%. The time needed to perform the task was mostly less than 10 min for monetary incentives less than US $1. Data quality validation was used in less than one-third of studies.

Our systematic review has advantages over previous ones on the same topic [5,10]. The systematic review conducted by Ranard et al in March 2013 described the scope of crowdsourcing in health and medical research but included only 21 articles [5]. The narrative review conducted by Swan described the use of crowdsourcing in health research studies up to 2011 [10]. Our mapping is more exhaustive—focused on health research but also health promotion and health care—and up-to-date. Many of our studies (80%) were published after the last search date of the Ranard et al’s systematic review [5]. This point highlights the increasing use of crowdsourcing in health during the last few years. Indeed, many health fields have since used crowdsourcing, with 20 medical fields identified in our systematic review compared with 8 fields in the Ranard et al’s study [5]. Moreover, crowdsourcing use is still growing, as shown by the 11 articles published in *Journal of Medical Internet Research* since our last search date, mainly involving a survey task (9/11, 82%) [217-227]. Our study has some limitations. First, we did not search the gray literature to identify some unpublished studies. However, the EMBASE search allowed us to identify 20 studies (10%) corresponding to conference abstracts. Second, we did not search Google Scholar because of the number of records found (about 30,000). Screening all these references would be extremely time-consuming for only 2 reviewers without using a crowdsourcing process. Third, we did not include studies related to biology, such as studies using the “Fold it” platform to solve protein-folding problems [228]. We did not consider this topic in our definition of health. Finally, we included only crowdsourcing performed via the internet. For example, we did not include studies in which the crowdsourced tasks were performed in a particular workshop without individual data collected online. Therefore, we may have underestimated the number of studies using crowdsourcing in health.

Every health category (promotion, research, and care) has a potential need for human computing power that crowdsourcing could fulfill to accelerate the process. Our systematic review, focusing on peer-reviewed papers, may have not captured some kinds of crowdsourcing. Studies recruiting crowd workers with social media platforms were few in our selection (12/202 studies [5.9%]). This type of recruitment seems less attractive than labor markets, although it is free and easier to use, perhaps because it is considered less reliable or used for purposes other than publication. Another way of exploiting social media data is under development, whereby tweets referring to a specific disease are analyzed as part of a health maintenance approach (eg, HIV in the Adrover et al’s study to identify adverse effects of drug treatment in tweets using crowdsourcing [229]). Considering health research, a fundamental aspect of this crowdsourcing is that it allows research to be performed *with* patients and not only *to* them or *on* them. However, studies with patients as crowd workers represented only 10% of our included studies, perhaps because the primary aim of collecting these data was not to conduct research with the data. Nevertheless, in 2013, the PatientsLikeMe platform [230] had more than 220,000 members sharing health data on more than 2000 diseases and conditions [231]. Using these data and conducting research with the data represent a great future challenge of mining crowd data and a real opportunity to collect large amounts of data on symptoms of diseases, drug efficacy, or adverse events to solve a wide range of health issues with a more real-life approach. Crowdsourcing also has potential in health promotion, especially preventive medicine, by taking it one step further. For example, specific tips in the form of slides or films could be added to the end of a Web-based survey about addiction to conduct a behavioral intervention, in addition to a simple survey. In some cases, data processing tasks may require thinking about a healthier lifestyle, for example, by suggesting healthier alternatives in addition to gathering information on the nutritional characteristics of packaged foods. Such crowdsourced tasks could be expanded to change dietary behaviors, exercise, or adherence to treatment. Finally, the combination of crowdsourcing and mobile health technologies could be the ultimate step in providing an ideal vehicle for behavioral interventions that can reach users in real time, in real life, without being resource-intensive.

Crowdsourcing allows for a large number of crowd workers to be mobilized in record time and at low cost. For instance, in Peabody et al’s study [158], experts completed 318 video ratings in 15 days, but crowd workers completed 2531 ratings in 21 hours. These crowdsourced resources might be further harnessed in a world of high health costs. Crowdsourcing also allows for speeding up innovations, when used in the form of collaborative scientific competitions—challenges—to solve diverse and important biomedical problems. Problem-solving was the fourth task we identified in terms of frequency, and only 7 challenges were individualized, perhaps because challenges are an emerging form of crowdsourcing, which should be more prominent in the next few years and lead to more publications [232]. In future, it will be necessary to facilitate and promote the use of this type of crowdsourced tasks in health research, given the amount of data to be considered (big data) and the complexity of medical issues that will require increasingly skilled and qualified individuals to resolve them.

As previously mentioned, crowdsourcing has many advantages: improved cost, speed, quality, flexibility, scalability, and diversity. However, some points that remain controversial include the impact of crowdsourcing on product quality or its unethical aspect. The first remaining potential concern of crowdsourced studies in health is the validity of their results. Some studies have assessed whether we should trust Web-based studies, and it appears that the data provided by internet methods have at least as good quality as those provided by traditional paper-and-pencil methods [233]. In our review, for data processing tasks, 36/202 feasibility studies (17.8%) compared crowd workers’ performance with that of an expert group considered as reference. These studies mainly considered surgical skills evaluation (15/36, 42%) and parasite identification in infectious diseases (4/36, 11%). At each time, the performance of crowd workers was similar to that of the reference group. However, because the participation is anonymous and compensated, participants may provide unsatisfactory quality data. In our review, 54/202 studies (26.7%) reported using data quality validation. Several types of validation techniques were found, from inserting random questions with known answers into the task, to screening for crowd workers who were incorrectly marking answers (31/54, 57%) and to comparing responses among multiple crowd workers to discard outliers (16/54, 30%). The second concern is its unethical aspect: Amazon MTurk is a bargain for researchers but not for crowd workers [234]. Indeed, many MTurk tasks are completed by a small set of workers who spend long hours on the website, many with low income.

A detailed description of the crowdsourcing logistics in the Methods section and all the characteristics of the crowd workers (population of the study) should be provided in high-quality research, even if its importance depends on the type of study. In cases of surveying and surveillance or monitoring studies related to illness, crowd workers’ characteristics need to be precisely described to better interpret the study findings and to judge the external validity. In cases of data processing and problem-solving, crowd workers’ characteristics also need to be reported to allow reproducibility of studies and to select more quickly and more easily the best population of crowd workers for a future similar study. In our review, the lack of details of crowd workers’ characteristics in one-third of the included studies impedes the interpretation of results of these studies. Rather than being a virtually infinite subject pool, crowd workers are far less diverse than was previously thought. As we found, although crowd workers should be recruited from all over the world, 61% were actually recruited nationally, mainly the United States (89%). Previously, crowd workers were mainly young, urban, and single and more often had postsecondary education [6]. In our review, the median age of crowd workers was 34 years, 55% were men, and half reported a high level of education. Therefore, logistics of crowdsourcing and crowd workers’ characteristics must be reported, and standardized guidelines on crowdsourcing metrics that needed to be collected and reported could be useful to improve the quality of such studies.

### Conclusions

Crowdsourcing appears to be a trendy, efficient, competitive, and useful tool to improve health actions, whether in preventive medicine, research, or care. Its use in health is increasing, particularly in public health, psychiatry, surgery, and oncology. Crowdsourcing allows for access to a large pool of participants, saves time to collect data, lowers costs, and speeds up innovations. Each health field could benefit from some tasks that could be crowdsourced to facilitate advances in research. To optimize the use of crowdsourcing in health, the logistics of crowdsourcing and crowd workers’ characteristics must be reported.

## Appendix

Multimedia Appendix 1 Protocol of the systematic review.

Multimedia Appendix 2 Search terms for MEDLINE and EMBASE (March 30, 2016).

Multimedia Appendix 3 Search strategy for ClinicalTrials.gov.

Multimedia Appendix 4 Details of the health fields considered.

Multimedia Appendix 5 Flow diagram of selection of studies applying crowdsourcing in health.

## Abbreviations

MTurk: Amazon Mechanical Turk
