# Supplementary material for: Mapping of Crowdsourcing in Health: Systematic Review
Source: J Med Internet Res. 2018 May 15;20(5):e187. doi: 10.2196/jmir.9330 (PMC5974463; doi:10.2196/jmir.9330)
Supplement: Multimedia Appendix 1 [file jmir_v20i5e187_app1.pdf]

Multimedia Appendix 1. Protocol of the systematic review.

- We uploaded a prespecified protocol to a publicly accessible institutional Website (http://www.clinicalepidemio.fr/protocols/).
