# Supplementary material for: Mapping of Crowdsourcing in Health: Systematic Review
Source: J Med Internet Res. 2018 May 15;20(5):e187. doi: 10.2196/jmir.9330 (PMC5974463; doi:10.2196/jmir.9330)
Supplement: Multimedia Appendix 2 [file jmir_v20i5e187_app2.pdf]

Multimedia Appendix 2. Search terms for MEDLINE and EMBASE (March 30th, 2016).

MEDLINE

**#1**	**"crowdsourcing"[MeSH Terms] OR crowdsource[tiab] OR crowdsourced[tiab] OR crowdsourcers[tiab] OR crowdsources[tiab] OR crowdsourcing[tiab] OR crowd-source[tiab] OR crowd-sourced[tiab] OR crowd-sourcing[tiab] OR crowdworker[tiab] OR crowdworkers[tiab] OR "crowd science"[tiab] OR "crowd-based"[tiab] OR crowds[tiab]**	**N= 949**
**#2**	**"Mechanical Turk"[tiab] OR "Mturk"[tiab] OR "crowdflower"[tiab] OR “foldit”[tiab]**	**N= 229**
**#3**	**“citizen science”[tiab] OR “citizen scientist”[tiab] OR “citizen scientists”[tiab]**	**N= 270**
**#4**	**Microtask[tiab] OR "online task"[tiab]**	**N= 19**
**#5**	**#1 OR #2 OR #3 OR #4**	**N= 1 363**

EMBASE

**#1**	**crowdsource OR crowdsourced OR crowdsourcers OR crowdsources OR 'crowdsourcing'/exp OR 'crowd source' OR 'crowd sourced' OR 'crowd sourcing'/exp OR crowdworker OR crowdworkers OR 'crowd science' OR 'crowd-based' OR crowds AND [embase]/lim**	**N = 705**
**#2**	**'mechanical turk' OR 'mturk' OR 'crowdflower' OR ‘foldit’ AND [embase]/lim**	**N = 164**
**#3**	**'citizen science' OR 'citizen scientist' OR 'citizen scientists' AND [embase]/lim**	**N = 177**
**#4**	**microtask OR 'online task' AND [embase]/lim**	**N= 12**
**#5**	**#1 OR #2 OR #3 OR #4**	**N= 991**
