# Supplementary material for: Mapping of Crowdsourcing in Health: Systematic Review
Source: J Med Internet Res. 2018 May 15;20(5):e187. doi: 10.2196/jmir.9330 (PMC5974463; doi:10.2196/jmir.9330)
Supplement: Multimedia Appendix 3 [file jmir_v20i5e187_app3.pdf]

Multimedia Appendix 3. Search strategy for ClinicalTrials.gov.

ClinicalTrials.gov: https://clinicaltrials.gov/
Advanced search:
Other terms: “crowdsourcing”
First posted: up to 03/30/2016.
