# Supplementary material for: Mapping of Crowdsourcing in Health: Systematic Review
Source: J Med Internet Res. 2018 May 15;20(5):e187. doi: 10.2196/jmir.9330 (PMC5974463; doi:10.2196/jmir.9330)
Supplement: Multimedia Appendix 4 [file jmir_v20i5e187_app4.pdf]

Multimedia Appendix 4. Details of the health fields considered.

- We included “public health” studies referring to:
  - any addiction (alcohol, tobacco, drug, food, social media);
  - high-risk behaviors for specific diseases;
  - monitoring of transmissible diseases;
  - public knowledge in medical fields;
  - public opinion about hospitals, health practitioners or health management
  - health communication.
- We considered “health literacy” studies in which crowd workers had to extract and curate key biological information from the literature into structure databases. This corresponds to the term “biocuration” used by Khare*.
- The field of “Medical education” referred to studies in which crowd workers had to solve medical problems or assess methods of medical learning.

***** Khare R, Good BM, Leaman R, Su AI, Lu Z. Crowdsourcing in biomedicine: challenges and opportunities. Brief Bioinform. 2016;17(1):23–32.
