# Supplementary figures and images for: Mapping of Crowdsourcing in Health: Systematic Review
Source: J Med Internet Res. 2018 May 15;20(5):e187. doi: 10.2196/jmir.9330 (PMC5974463; doi:10.2196/jmir.9330)

Multimedia Appendix 5. Flow diagram of selection of studies applying crowdsourcing in health.

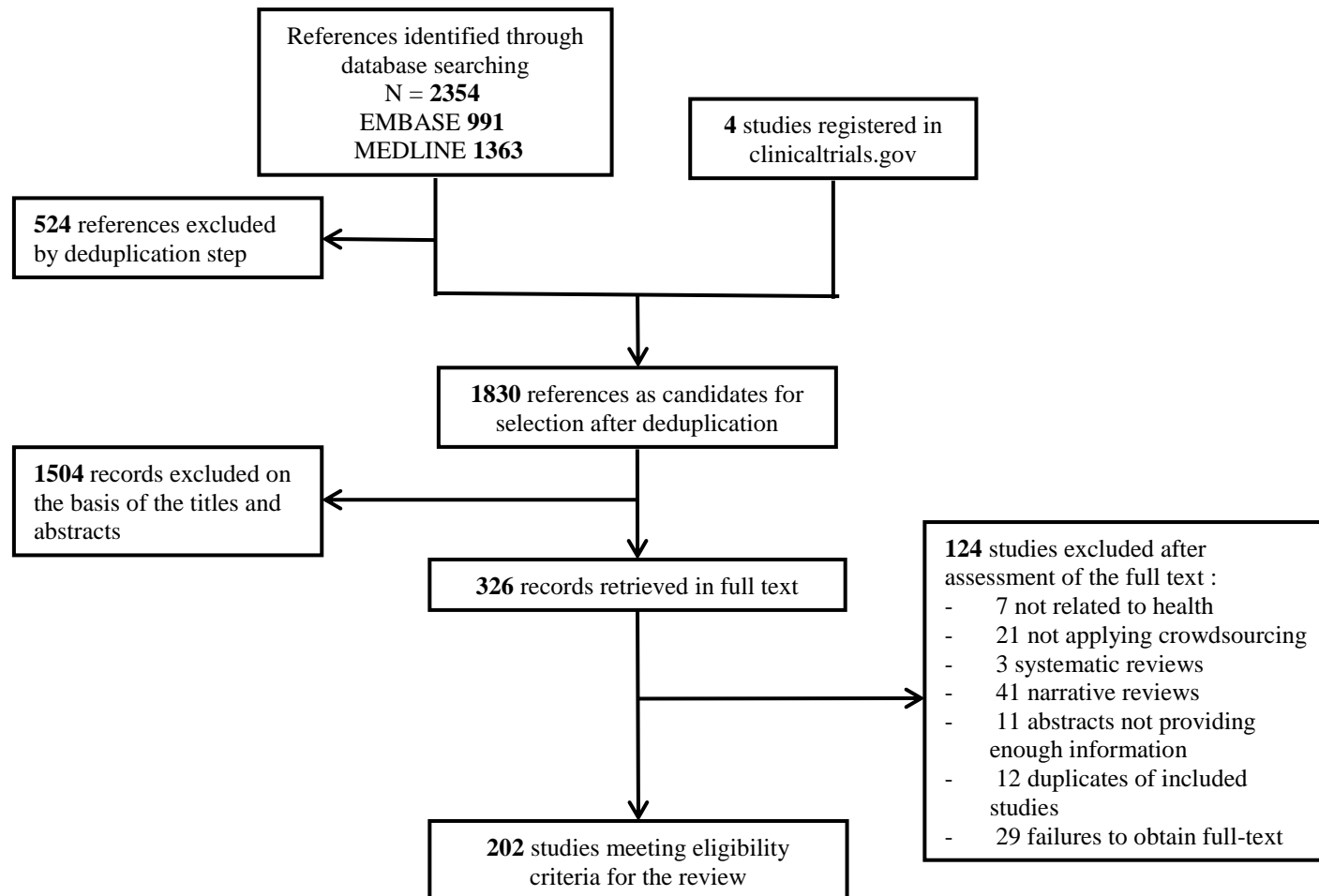

Supplement: Multimedia Appendix 5 [file jmir_v20i5e187_app5.pdf]
